# Sleep Monitoring Based on a Tri-Axial Accelerometer and a Pressure Sensor

**DOI:** 10.3390/s16050750

**Published:** 2016-05-23

**Authors:** Yunyoung Nam, Yeesock Kim, Jinseok Lee

**Affiliations:** 1 Department of Computer Science and Engineering, Soonchunhyang University, Asan 336-745, Korea; ynam@sch.ac.kr; 2 Department of Civil & Environmental Engineering, Worcester Polytechnic Institute, Worcester, MA 01607, USA; yeesock@wpi.edu; 3 Department of Biomedical Engineering, Wonkwang University School of Medicine, Iksan, Jeonbuk 570-749, Korea

**Keywords:** health care, sleep monitoring, sleep apnea, pressure sensor, heart rate variability, breathing, sleeping pose, polysomnography

## Abstract

Sleep disorders are a common affliction for many people even though sleep is one of the most important factors in maintaining good physiological and emotional health. Numerous researchers have proposed various approaches to monitor sleep, such as polysomnography and actigraphy. However, such approaches are costly and often require overnight treatment in clinics. With this in mind, the research presented here has emerged from the question: “Can data be easily collected and analyzed without causing discomfort to patients?” Therefore, the aim of this study is to provide a novel monitoring system for quantifying sleep quality. The data acquisition system is equipped with multimodal sensors, including a three-axis accelerometer and a pressure sensor. To identify sleep quality based on measured data, a novel algorithm, which uses numerous physiological parameters, was proposed. Such parameters include non-REM sleep time, the number of apneic episodes, and sleep durations for dominant poses. To assess the effectiveness of the proposed system, three participants were enrolled in this experimental study for a duration of 20 days. From the experimental results, it can be seen that the proposed monitoring system is effective for quantifying sleep quality.

## 1. Introduction

Sleep quality is necessary for a healthy life; sleep comprises approximately one-quarter of the human life span, and plays an important role in resting the brain. Several indicators can be used to describe sleep disturbances or sleep disorders, including: sleep latency; the number and duration of nocturnal awakenings; the total sleep time; changes in the number and rhythms of particular sleep stages, such as rapid eye movement (REM) state and non-rapid eye movement (non-REM) state; and recurring nights of sleep disruption, over one week or one month. These indicators can be measured by monitoring different physiological parameters during sleep. It is very important to monitor these parameters daily in a given individual while they sleep [1,2]. Respiration, heart rate (HR), temperature, body movements, and blood pressure are the main physiological parameters that indicate sleep quality. HR and respiration are known to vary greatly during sleep [3] and have a close relationship with each sleep stage [4] since the autonomic nerve system significantly affects HR and respiration. In particular, respiration is considered the most important parameter of physiological data because it clearly indicates sleep disorders such as snoring and sleep apnea.

In addition, body movement is also linked to sleep level, such as non-REM and REM sleep [5]. Such sleep levels can be estimated by monitoring body movements during sleeping [6]. Rechtschaffen and Kales [7] proposed a sleep scoring standard. Sleep states consist of two general stages: REM and non-REM sleep. Non-REM sleep can be divided into a further four stages [8]. Typically, physical wellbeing is rejuvenated in REM sleep, followed by additional rest in non-REM sleep. When one of these two sleep phases is not achieved, a person may feel like they have had poor sleep. It is generally very difficult to avoid poor sleep quality when one of the following three requirements is absent: suitable sleep duration, a deep sleep, and a periodical sleep cycle of REM and non-REM sleep. Sleep quality [9] should be estimated using effective methods to evaluate these three requirements.

Various studies have been conducted regarding how to measure sleep states based on physiological information. In practice, self-rated questionnaires and sleep diaries are routinely used for the assessment of sleep quality. Among the questionnaires, the Pittsburgh Sleep Quality Index (PSQI) [5] has been widely used as a diagnostic instrument. The PSQI contains 19 self-rated questions which form seven component scores. Each component score ranges from 0 to 3. The sum of the subscale scores yields a global score of sleep disturbance between 0 and 21. Higher scores indicate more severe sleep disturbance. However, retrospective assessment of sleep quality is subjective to the individual, thus leading to a less reliable result.

Polysomnography (PSG) [10] is usually conducted at specialized centers or in hospitals, and is a standard approach for sleep monitoring and objective measurement of sleep quality. PSG involves recording multiple physiologic variables, including electro-encephalogram (EEG), electro-cardiogram (ECG), electro-myogram (EMG), and electro-oculogram (EOG) [11]. Such PSG data are scored by human examiners based on standardized criteria. The PSG recordings provide an accurate assessment of sleep architecture and quality. However, the high cost of PSG makes it impractical to implement it within a long-term sleep monitoring system. In addition, attaching many sensors to a subject’s body is considered intrusive, and may in turn disturb sleep. Consequently, the measured data may not accurately represent the sleep behavior of subjects. In addition, to confirm the diagnosis, PSG needs to be conducted using a large, complex system with support from doctors and other experts.

Another widely-adopted objective measuring device is the actigraph [1], which is a watch-like device containing motion accelerometers to measure limb movements. Actigraphy has been used for many medical research applications, typically for monitoring motion-related sleep disorders. Actigraphy has been used to study sleep–wake patterns for at least 30 years; *i.e.*, since Kupfer *et al.* [12] reported a significant correlation between wrist activity, EEG signals, and wakefulness in 1972. Sadeh *et al.* [13] concluded that normal subjects showed more than 90% correlation when comparing actigraphy data with PSG. By 1995, sufficient experimentation had been carried out to finally enable the Standards of Practice Committee of the American Sleep Disorders Association to support the use of actigraphy in evaluating certain aspects of sleep disorders, such as insomnia, circadian sleep–wake disturbances, and periodic limb movements.

Although only one physiological variable is measured, the advantage of actigraphy over PSG is that sleep and wakefulness can be recorded continuously over a period of weeks, or even longer. In other words, actigraphy provides a convenient way for long-term sleep-monitoring. However, the device is still considered intrusive in that some people may feel uncomfortable when they wear a wrist-watch type device during sleep. Additionally, actigraphy is not as effective when detecting certain sleep disorders that do not involve limb motion, but in which extensive pre- and post-signal processing are required. The portability of this system allows the patient to move freely. However, sometimes the patient can unconsciously remove the sensors during sleep, and attachment of sensors via a pressure sensor sheet imposes a physical and mental burden on the patient.

Static charge sensitive beds (SCSB) [14] can be also used to monitor patient respiration and HR during sleep. An unrestrained sleep monitoring system, using cameras, has been proposed to monitor these parameters. However, the procedure may still be considered as an invasion of privacy by some users, in whom its use would therefore not be suitable. For a non-invasive analysis of physiological signals (NAPS) [15], a sleep monitoring system that monitors patient respiration and body movements during sleep, via a pressure sensor, is proposed. In these systems, patient respiration and body movements can be monitored freely using sensors attached to the periphery of the bed. However, because it is not typically an easy task for people to exchange beds, effective installation of these systems at home can be challenging.

A nasal pressure recording technique presents a very promising tool for medical research, constituting a noninvasive method and permitting a better understanding of the underlying pathophysiologic abnormalities associated with sleep-disordered breathing. Aittokallio *et al.* [16] tried to obtain information on upper airways behavior of patients and health subjects. The apnea and hypopnea index are more relevant with diagnosis of obstructive sleep apnea syndrome (OSAS). Several kinds of airflow sensors are used to monitor respiratory airflow, such as a pressure sensor [17], hot-wire and hot-film sensors, an infrared thermography [18], and an ultrasonic flow meter [19]. Recently, fiber optic sensors have been proposed for monitoring of breathing rate, heart rate and body movement in [20,21,22]. Fiber Bragg grating (FBG) based sensors have been used for the simultaneous measurement of breathing rate and heart rate. However, its wavelength detection technology is too complex and expensive for sensor fabrication and instrumentation.

In this paper, a non-intrusive system for monitoring sleep quality is proposed to limit the concerns over privacy associated with video sensors. The proposed system was developed as a multimodality sensor fusion framework using a variety of sensors. Based on a pressure sensor and an accelerometer, data on motion, respiration, body activity, and HR can be extracted. The multimodal sensors directly extract features from the embedded mobile device, which sends physiological data to either home servers or a remote clinical monitoring center via an internet connection to determine sleep state. It has been shown in experimental studies that this proposed system is effective in detecting sleeping and waking states. The contributions of this paper are as follows: (1) a novel system is proposed involving integration of low-cost multimodality sensors and several methods for sleep condition monitoring and sleep quality measurements; (2) the proposed system effectively extracts key features from the sensing data and fuses information from different sensors for detection of waking from sleep. It is expected that the proposed system can replace existing standard methods (e.g., PSG or actigraphy) to measure sleep. Our system also constitutes an alternative approach, which has the potential to monitor sleep quality. It should be noted that the proposed system does not require the collection of video data, which is typically required by existing approaches. Furthermore, the proposed system does not require any input or analysis from trained experts to manually assign inference rules; and (3) the design is expandable and can be used with the proposed multimodality sensor fusion framework, allowing additional monitoring capabilities with other types of non-intrusive sensors.

The rest of the paper is organized as follows. In Section 2, the proposed system and its associated methodology are presented. The performance of the proposed system is evaluated using a 20-day field test in Section 3. The experiment includes sleep stage detection in different sleep cases. In Section 4, directions for future work are identified and the performance deterioration is discussed due to body motion artifacts. Finally, concluding remarks and the future directions of current research are given in Section 5.

## 2. Sleep Quality Monitoring System

### 2.1. System Architecture

**Figure 1:**
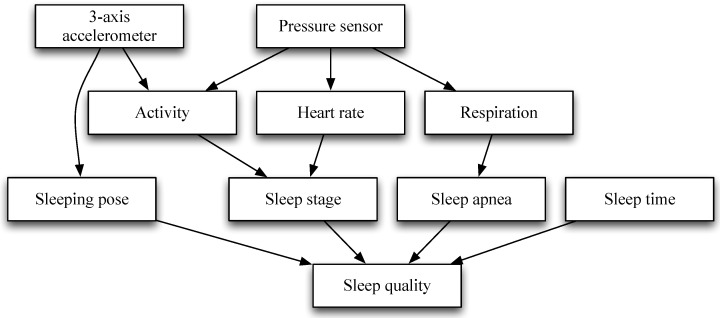
Process flow for sleep quality monitoring.

The process flow for sleep quality measurement is illustrated in Figure 1. Sensing data were collected from two different sensors and then transmitted via a ZigBee wireless connection to an external assistive recording mobile device and PC platform. Sleep pose and activity are determined by sensing data obtained from a three-axis accelerometer sensor. Heart rate and respiratory rate are estimated by sensing data obtained from a pressure sensor. Sleep stage is classified by activity and heart rate. Finally, sleep quality is calculated by using sleeping pose, sleep apnea, and sleep time.

### 2.2. Feature Extraction and Data Analysis

Spectral analysis for analyzing HR has been applied to various medical problems [23,24,25]. In this study, the raw data measured by the proposed system were grouped into two epochs and analyzed using an automated algorithm. After analyzing one group, the data were pre-processed using a bi-directional recursive filter to ensure a phase shift is not introduced. Once the data are smoothed, the peaks and troughs of each waveform can be found. The first involved simple derivative and threshold peak detection, while the second looked for changes in the direction of relative trough positions. By using this technique, HR was determined in a similar manner to that used for measuring the length of consecutive cardiac periods (R-R intervals) of ECG.

**Figure 2:**
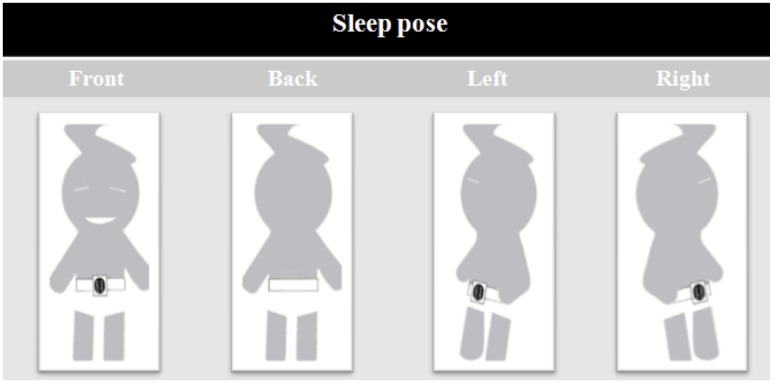
Sleeping postures.

The variability of HR and respiratory signals, both derived from ambulatory ECG recordings, can be analyzed using power spectral analysis. Based on the average amplitude obtained from a sensor, a one-minute window of breathing data was normalized as a post-processing method. The average amplitude of the breathing data was then used to determine the expected amplitude for breathing, and then a threshold for variable amplitudes was determined to detect breaths. In cases where there were signals from slight movement artifacts, or changes in breathing patterns, the signal amplitude was analyzed to clarify what was considered as a full breath. The clinical and research definitions of breathing events during sleep are used to provide a physiological base from which to determine the experimental criteria for defining apneaic episodes and arousals. As a possible classification of arousal, an amplitude over 140%, of a signal containing a minimum amount of postural movement, was considered. As a possible classification of apnea, an amplitude under 75%, with gaps in the signal and minimal postural movements, was considered. Sleeping pose was defined according to unconscious motions during sleep, such as rotational body movements. A change in sleeping pose was defined as a series of trunk motions from a static state to a new static state due to rotational motions during sleep. Movement of the limbs alone was not regarded as a change in sleeping pose. The body positions were grouped into four categories: front, back, left, and right, as shown in Figure 2.

**Figure 3:**
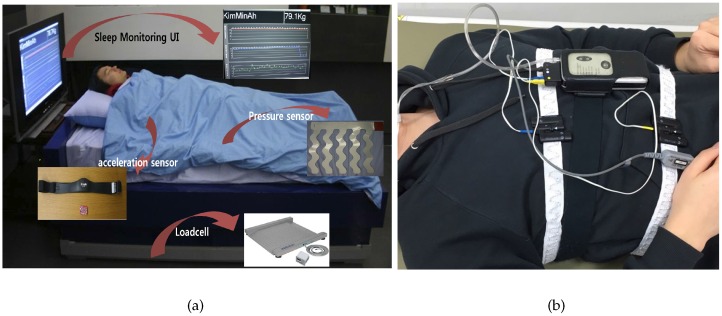
Our test-bed environment, (**a**) test-bed; (**b**) Polysomnography.

As shown in Figure 3, the system begins recording when the user goes to bed and stops recording when they wake up. To evaluate our system, an Embletta portable diagnostic system (PDS, Medcare, Reykjavik, Iceland) was used, as shown in Figure 3b. The Embletta portable diagnostic system is widely used for sleep apnea screening in clinical practice. The system uses a digital three-channel recording device to measure airflow through the nasal cannula, connected to a pressure transducer, with oxygen saturation plus both respiratory and abdominal movements measured via built-in effort and body position sensors. Thoracic and abdominal effort was measured using two belt sensors. Saturated oxygen in arterial blood (SpO${}_{2}$) was recorded using digital pulse oximetry (sampling frequency of 1 s). Respiratory event detection and oximetry analysis were performed manually. The sleep quality was classified into REM $S_{\mathrm{REM}}$ and non-REM $S_{\mathrm{non}-\mathrm{REM}}$ sleep. REM sleep is the lightest type of sleep, while non-REM sleep is the deepest type.

**Figure 4:**
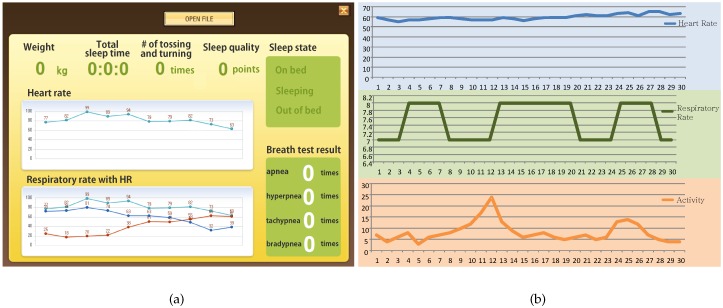
Screenshots of the user interfaces and examples of signals obtained from the sleep quality monitoring system. Screenshots of user interfaces in the sleep quality monitoring system. (**a**) screenshots of user interfaces in the sleep quality monitoring system; (**b**) example signals obtained from the sleep quality monitoring system.

Figure 4a shows the user interface (UI) of the sleep quality monitoring system. The developed application provides data on weight, total sleep duration, the degree of tossing and turning, sleep quality, sleep state, HR, respiratory rate, and breath test results. Figure 4b shows an example of the signals obtained from the sleep quality monitoring system. It can be seen that, while the HR ranged from 55 to 65 beats per minute (BPM), the respiratory rate ranged from seven to eight breaths per minute during sleep.

**Figure 5:**
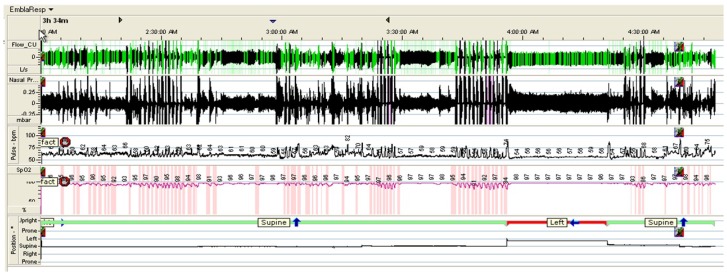
Example of signals obtained from the PSG.

Figure 5 shows an example of the signals obtained from the PSG, including respiratory signals, with airflow through a nasal cannula connected to a pressure transducer, HR and SpO${}_{2}$, as well as and body position.

In [8,26], it has been shown that the heart rate variability (HRV) spectral parameters exhibit significant differences between the different sleep stages and, in particular, they seem to discriminate well between REM and non-REM sleep. In this paper, sleep stage is classified as REM or non-REM sleep based on [27]. The sleep stage classification $S_{\mathrm{stage}}$ is as follows:

(1) $$\begin{array}{c} S_{\mathrm{stage}}={\left\{\begin{array}{c} S_{\mathrm{Non-REM}},\;\;\mathrm{if}\;T_{Amin}\leq A_{i}\leq T_{Amax}\;and\;T_{Hmin}\leq A_{i}\leq T_{Hmax},\\ S_{\mathrm{REM}},\;\;\;\;\;\;\mathrm{otherwise}, \end{array}\right.}_{} \end{array}$$

where $T_{Amin}$ is the minimum activity threshold value, $T_{Amax}$ is the maximum activity threshold value, $A_{i}$ is the activity value, and $H_{i}$ the HR value.

Sleep apnea $S_{\mathrm{apnea}}$ is shown by respiration amplitudes under 75% or gaps in the signal accompanied by minimal postural movement, as follows:

(2) $$\begin{array}{c} S_{\mathrm{apnea}}={\left\{\begin{array}{c} True,\;\;\mathrm{if}\;R_{i}\leq T_{R}\;\mathrm{and}\;R_{ti}-R_{ti+1}\leq T_{tR},\\ False,\;\mathrm{otherwise}, \end{array}\right.}_{} \end{array}$$

where $T_{R}$ is the respiration threshold value, $R_{i}$ is the respiration value, $R_{ti}$ is the respiration *i* value, and $T_{tR}$ is the threshold time.

Sleep quality $S_{\mathrm{quality}}$ can then be calculated using Equation (3). The sleep quality is determined from three parameters: non-REM sleep time, the number of apneaic episodes, and the total duration of the subject’s dominant sleeping pose. Each parameter has a different impact factor, which is empirically determined: *α* = 0.5, *β* = 0.3, and *γ* = 0.2, which did not seem optimal in the empirically derived weights. We hope that other laboratories will be able to evaluate these parameter in a diversity of samples. Furthermore, controlled comparisons between different approaches are needed to assess where improvements are needed in electronic design and a sleep monitoring system. The preferred sleeping pose is determined as the pose that was adopted for the longest duration, over non-sleep time:

(3) $$\begin{array}{c} S_{\mathrm{quality}}=\left(\frac{S_{D}}{S_{T}}\cdot \alpha \right)+(100-A_{N})\cdot \beta +\left(P_{T}\cdot \gamma \right) \end{array}$$

where $S_{T}$ is the sleep duration, $S_{D}$ is the difference between the sleep time and REM sleep time, $A_{N}$ is the number of apneaic episodes, $P_{T}$ is the duration of the preferred sleeping pose, and *α*, *β*, *γ* are impact factors. The sum of *α*, *β*, and *γ* is 1. In the experiments, the sleep state is classified into REM and non-REM sleep states, based on the patients’ activities and HRs.

## 3. Experimental Results

### 3.1. Experimental Environments

**Figure 6:**
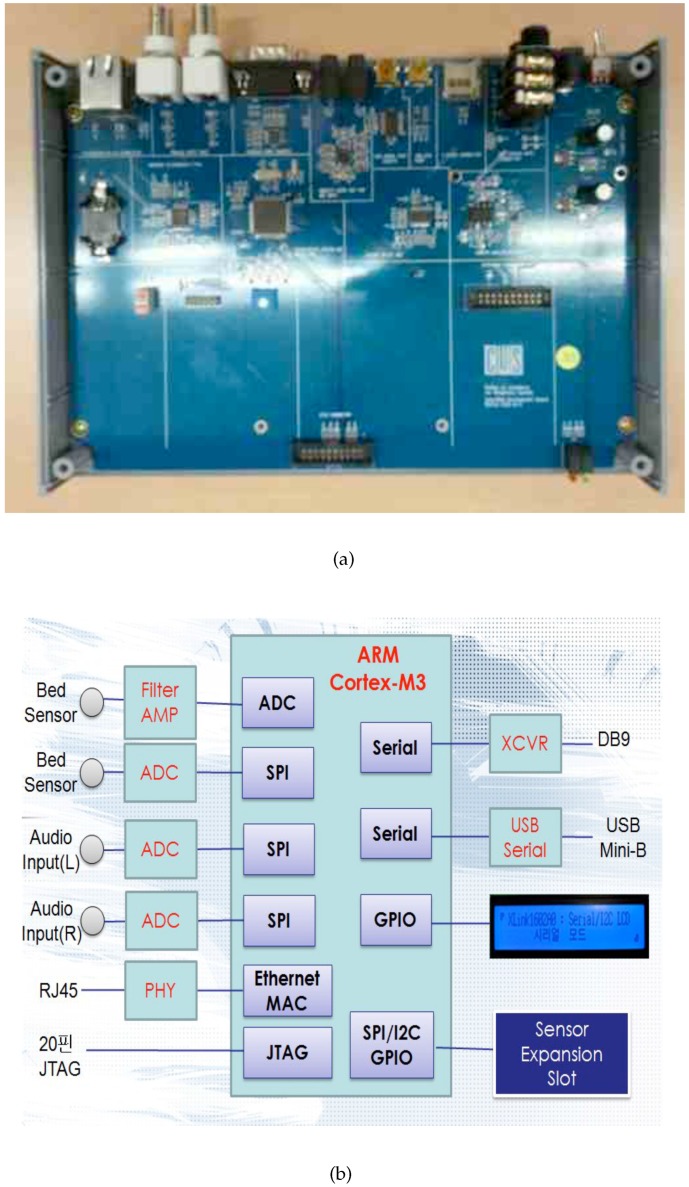
Analog-to-digital converter (ADC) board. (**a**) ADC board; (**b**) architecture of ADC board.

To evaluate the sleep quality monitoring system, an accelerometer and a wearable sensing belt were used to obtain continuous data on each participant’s sleeping pose, as shown in Table 1 and Figure 6. A pressure sensor was used to obtain the user’s physiological data, including respiration, HR, and activity rate. The pressure sensor was installed in a conventional bed. The sleep–wake cycle was determined based on respiratory signals acquired through a pressure bed sensor.

**Figure 7:**
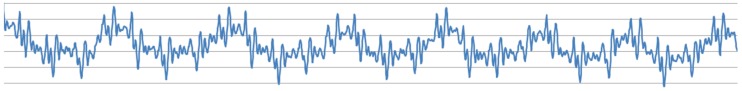

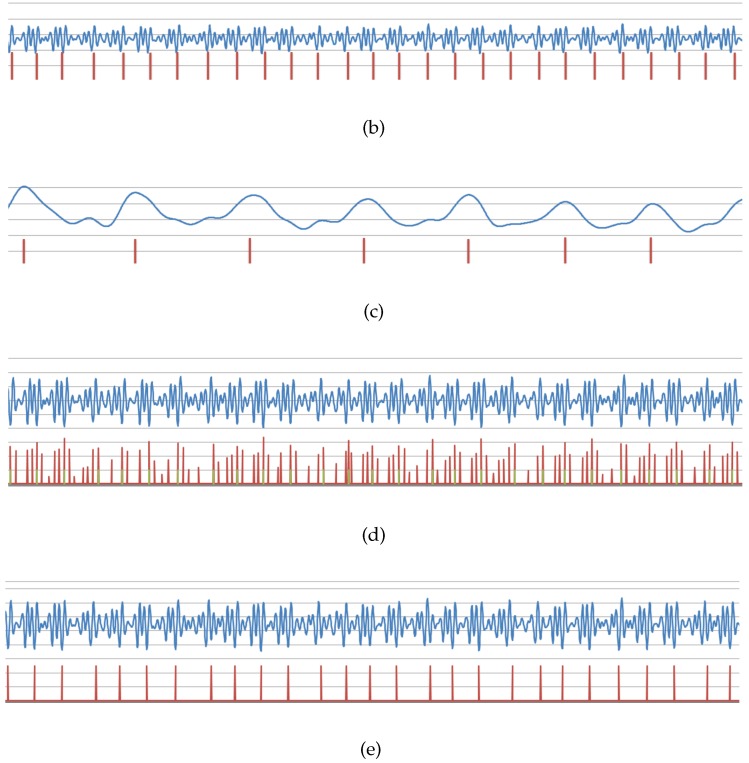
Heart rate variability and respiratory rate. (**a**) Raw data obtained from ADC (100 samples/s, 12-bit resolution); (**b**) Filtering data using an infinite impulse (IIR) filter for estimating the heart rate; (**c**) Filtering data using an infinite impulse (IIR) filter for estimating the respiratory rate; (**d**) Heart peak obtained from filtering data after noise reduction; (**e**) Selected heart peak.

The subjects used for the experiments were 10 college/graduate student volunteers, ranging in age from 20 to 30 years and including one female. The subject’s respiration, HR, activity, and sleep poses were measured overnight using the system. In addition, a digital video camera recorded the subject’s respiration, HR, activity, and sleeping poses for use as reference data. Figure 7 provides an example of heart and respiration data for an entire night from one subject. From the figure, the signals obtained from a pressure sensor have many noises. Although it is difficult to extract the QRS complex features, the HR can be estimated by using detecting the R waves from the filtered data and measuring the RR interval sequence in a fixed time window. HR is estimated by using a 3 s sliding window. In the experiments, the HR is estimated every second and found 2–4 times errors in 1 min.

**Figure 8:**
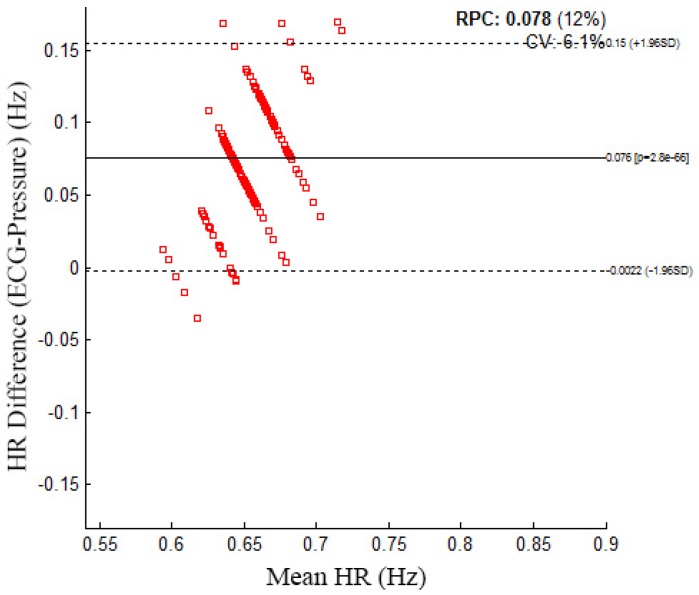
Bland–Altman plot with a mean difference of 0.076 that shows the limit of agreement of 95% (dashed lines are mean differences ± the limit of agreement) between the continuous heart rate (HR) of pressure signal and its corresponding electro-cardiogram (ECG) signal.

**Figure 9:**
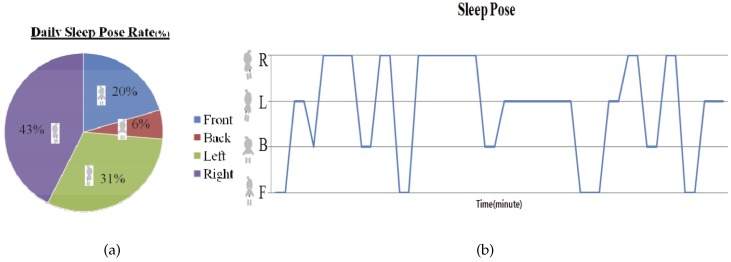
Results of the sleeping pose rate and sleeping pose detection. (**a**) Sleeping pose rate; (**b**) Sleeping pose detection.

Figure 8 shows the Bland–Altman plot for the mean HR data from the pressure sensor and the ECG. The Bland–Altman plot shows a mean difference of 0.076 and that most of the data are within the 95% confidence intervals. An example sleeping pose and individual daily sleeping pose rate is illustrated in Figure 9. The daily sleeping pose rate is used to determine the individual dominant sleeping pose.

### 3.2. Results

**Figure 10:**
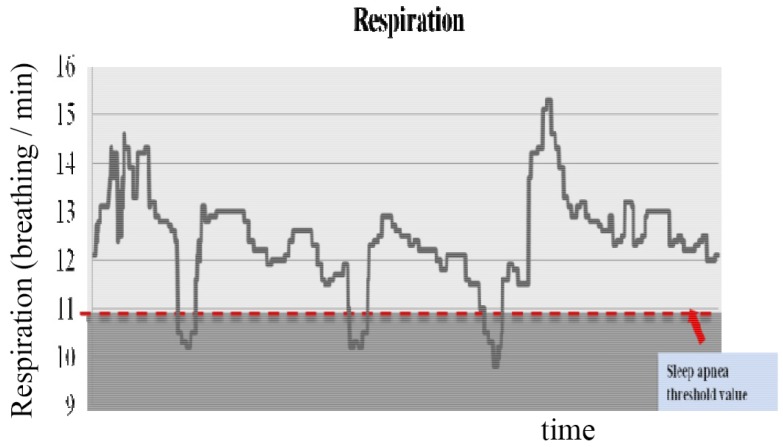
Sleep apnea detection.

In the experiment to monitor sleep quality, Figure 10 illustrates an example of respiration signals from midnight to 2:00 a.m. The red dotted line shows the threshold value for sleep apnea. The threshold value is determined from the amplitude of the respiration signal (below 75%).

**Figure 11:**
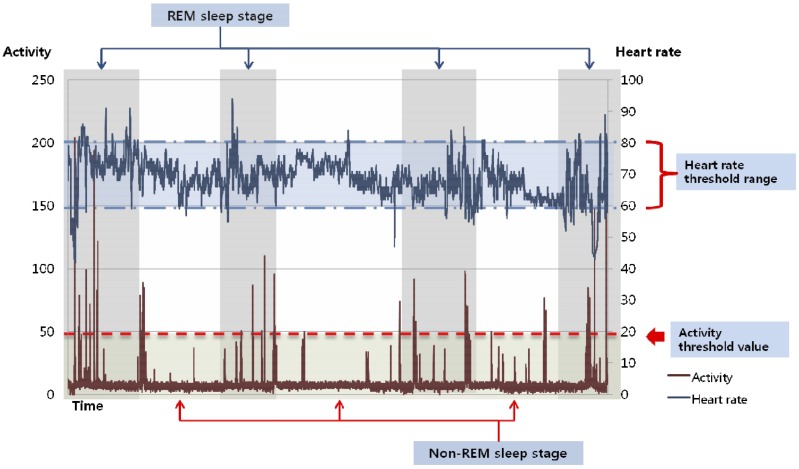
Results of the sleep stage classification.

Figure 11 shows the sleep stage classified by the proposed method. The sleep stage is calculated from the HR and the activity values. The red line shows the subject’s activity and the blue line shows the HR signal from midnight to 2:00 a.m. Data collected over 20 days is used to validate the effectiveness of the proposed system, with the remaining data randomly partitioned into training and testing sets for each trial. Table 2 shows the experimental results regarding sleep quality. One-way ANOVA was performed to compare our approach with PSG. Bonferroni *t*-test analysis was performed if significant statistical differences were found. Values of *p* < 0.05 were considered statistically significant.

The average sleep time was approximately 7 h, where the duration of sleep of subject E was the longest, and that of subject D was the shortest. It can also be inferred that subject E experienced the highest quality sleep compared to the other subjects because, for this subject, other related factors had a higher rating than did those for the other subjects. The number of apneaic episodes and total time spent in the dominant sleeping pose are considered more important than the total sleep time. Subject F had the lowest sleep quality because this subject also suffered from a major depressive disorder. These results showed that the proposed sleep quality monitoring system has very high efficiency and reliability.

## 4. Discussion

The parameters proposed in our sleep quality monitoring system have not been considered in traditional research. In the future, the relationship between sleep quality and other physiological signals will be studied to further improve the performance of the proposed sleep quality equation. Furthermore, the system will be incorporated into a dynamically retainable system to improve adaptability to the needs of individual users. It is expected that the improved robustness of the proposed multimodal sensor fusion framework can be extended to other types of non-intrusive sensing systems.

**Figure 12:**
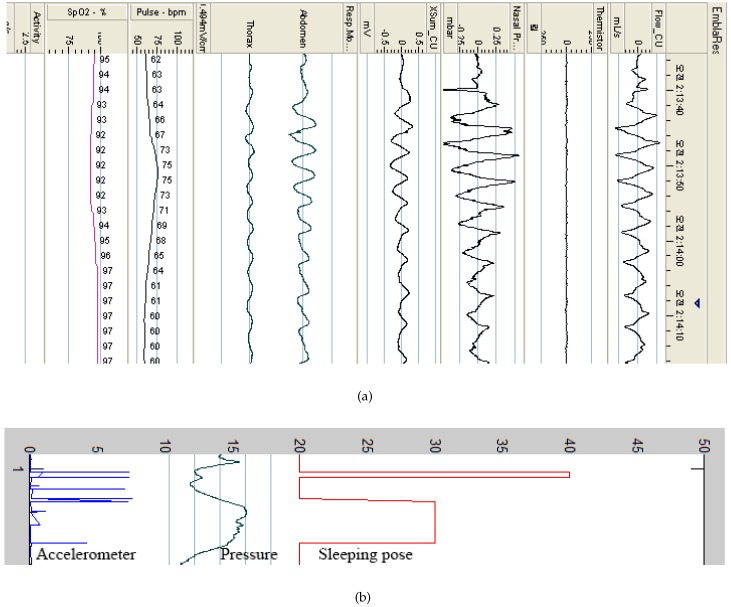
Example of signals obtained from sensors in the presence of motion artifact. (**a**) Example of signals obtained from the PSG in the presence of motion artifact; (**b**) Example of signals obtained from an accelerometer sensor and a pressure sensor in the presence of motion artifacts.

During sleep, every movement of the body will affect the signal. When a patient moves on a bed, it is difficult to estimate HR and RR using signals obtained from only a pressure sensor. Figure 12 shows an example of the signals obtained from sensors in the presence of motion artifact. From the figure, signals obtained from the PSG can be reconstructed for estimating HR and RR, signals obtained from a pressure sensor cannot be reconstructed due to motion artifacts.

## 5. Conclusions

In this paper, a sleep quality monitoring system has been proposed. The sleep quality monitoring system determined the sleeping pose, sleep state, REM sleep stage and non-REM sleep stage cycle using a three-axis accelerometer and a pressure sensor, without the need for a large system, such as the PSG. In addition to such sleep stages, the proposed system can measure sleep quality by estimating the depth of sleep, the number of apneaic episodes and the periodicity. The proposed system also analytically calculates sleep quality and detects early symptoms of sleep-related disorders and appropriate responses to treatment. The experimental results demonstrated that the proposed system is effective in measuring the physiological factors of sleep quality. Furthermore, it was also observed that the estimations used for the proposed sleep quality equation were significantly reliable. This is because many physiological parameters are incorporated into the equation, including non-REM sleep time, the number of apneaic episodes, and the total time spent in the dominant sleeping pose.

## Figures and Tables

**Table 1:** Specifications of sensors.

Sensor		Specifications
three-axis accelerometer	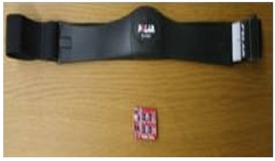	Size: 5 cm, weight: 500 g, consumption current: 0.6 mA, resolution: 60 Hz, MSP430 micro controller for a micro controller (MCU): 16 bit reduced instruction set computer (RISC)
pressure	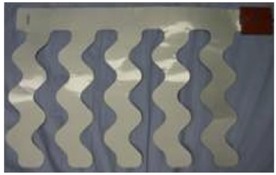	Size: 40 cm × 40 cm, weight: 300 g, sensor type: film, operating temp: from −40 ${}^{\circ }$C to +50 ${}^{\circ }$C, sensitivity: 25–250 pc/n, operating force range: >100 N/cm${}^{2}$

**Table 2:** Results of sleep quality.

Subject	Total Sleep Time (Hour)	The Number of Sleep Apnea (Ours)	The Number of Sleep Apnea (PSG)	The Number of Sleep State Change (Ours)	The Number of Sleep State Change (PSG)	Sleep Quality	Dominant Sleeping Pose
A	7.1	16.3	16.6	5.6	5.8	75.77	Right
B	6.8	12.7	12.2	4.8	4.4	81.54	Front
C	7.6	13.6	13.2	5.2	5.6	77.57	Right
D	5.1	0	0	4	4	87.44	Right
E	10	0	0	3	3	120.3	Front
F	7	0	0	16	16	74.17	Back
G	7	5	5.2	6	6	77.66	Right
H	5.6	0	0	2	2	86.67	Front
I	7	2	2.2	3	3	104.75	Front
J	7	16	16.2	3	3	85.81	Front
